# Real-time Patient Experience Surveys Lead to Better Scores

**DOI:** 10.5811/westjem.18713

**Published:** 2025-06-25

**Authors:** Keith Willner

**Affiliations:** Geisinger Wyoming Valley Medical Center, Department of Emergency Medicine, Wilkes-Barre, Pennsylvania

## Abstract

**Introduction:**

The patient satisfaction survey is a controversial fixture of modern emergency care. Patients who are satisfied are more likely to adhere to the treatment plan and less likely to pursue legal action. However, the current surveys are susceptible to recall bias. This study uses an analysis of data collected in a separate study to assess how patients rated their physicians’ care when asked key questions in person by a trained volunteer versus in the Doctors section of the Press Ganey (PG) survey.

**Methods:**

This was an analysis of prospectively collected data obtained in a separate study evaluating how patients experience their emergency care when learners are present. Trained medical student volunteers administered the survey to a convenience sample of patients slated for discharge at a single, community, tertiary-care hospital emergency department (ED) for a total of 12 weeks between June–October 2022. We compared this with the hospital’s PG data for the questions on which the survey was based.

**Results:**

A total of 625 patients were approached over the study period with 313 agreeing to participate (response rate 50.1%). There were 8,460 patients discharged from the ED during those times (overall rate 3.70%). During the contemporaneous PG study quarter, the ED received 266 responses during the shifts for which the study enrolled patients, of a total 8,460 discharged from the ED during those times (response rate 3.14%). All key questions favored the in-person survey vs mailed PG survey: “I felt informed” score 79.2 (262) vs 75.6 (265), *P* = .02; “I felt like my [doctor] took time to listen” 85.0 (261) vs 79.6 (266), *P* = .05; and “satisfaction with care team” 83.0 (263) vs 74.7 (265), *P* = .0013.

**Conclusion:**

This study shows higher satisfaction scores with an in-person survey. There was also a dramatically improved response rate compared with mail in PG forms, suggesting less recall bias. An absolute 5-point difference in PG score could lead to a relative 30-point change in percentile rank. This was a limited, single-site study whose results are hypothesis-generating but suggest a new pursuit for administrations seeking to improve their scores and possibly better understand patients’ experience of their care.

## INTRODUCTION

### Background

The patient satisfaction survey is an ubiquitous feature of modern medical practice and healthcare administration. As practitioners we want our patients to be satisfied with their care. Multiple studies have demonstrated that satisfied patients are more likely to complete the recommended treatment course and less likely to pursue litigation.^1^,^2^,^3^ Whether increasing satisfaction leads to better outcomes is controversial.^1^ Furthermore, hospital reimbursement and revenue generation are tied to these scores.^1^

The default industry standard is the Press Ganey (PG) survey, which is mailed to patients after their visit to the emergency department (ED). Their large database allows comparison within the hospital between physicians, over time, and between peer institutions. However, this is a survey tool, and response rates are generally poor; additionally, since the form is sent through the mail, responses are subject to recall bias.^4^,^5^ Some studies suggest responses to these surveys are biased against female and minoritized physicians vs their counterparts.^5^,^6^ Because many departments use these results for promotion and compensation, mitigating bias is paramount.

### Importance

All stakeholders want their patient satisfaction scores to reflect the actual patient experience of their care without bias, while keeping them as high as possible. A survey design that minimizes bias and results in an increased response rate has the potential to help administrators make changes and allow for more robust feedback to individual clinicians. If changing the delivery of the survey can do that at minimal cost, this is an avenue administrators may wish to pursue.

### Goals of This investigation

Understanding the limitations of the current industry standard survey tools, we investigated whether there might be a better way to capture our patients’ experience of their care. Our research team, which was conducting a study to explore how the presence of learners affects perceptions of care, hand-delivered the survey to each patient. We performed a reanalysis of that in-person survey data and compared it to our institution’s PG scores. Our initial hypothesis was that surveys administered in person would return better scores with a higher response rate with respect to the three satisfaction questions related to the corresponding “Doctors” questions on the PG survey.

## METHODS

### Study Design, Setting, and Participants

This study was based on data from a single, suburban Level I trauma center community ED averaging over 50,000 visits annually. Patients eligible to be enrolled in the parent study were adults ≥18 years of age who were slated for discharge from the ED. For reasons of informed consent, they could not have been seen for a behavioral health or substance use diagnosis and could not have been incarcerated at the time of enrollment. Patients were also excluded if they were made a trauma alert, triaged as Emergency Severity Index (ESI) level 1, or were seen by an advanced practice practitioner (APP). This was to prevent confounding with multiple participants on the care team to prevent misperception of a learner.

Our PG data does not include individual level characteristics, and it is possible some patients returned both surveys. Additionally, patients seen by advanced practitioners, triaged to ESI level 1 and discharged, and seen as a trauma alert and subsequently discharged all would be eligible to receive a PG survey, but would not have completed the parent research survey. The survey was administered by a trained research assistant (RA), not part of the care team, who obtained consent for inclusion in the parent study at the time of enrollment. The survey was delivered electronically on an iPad, using REDCap electronic data capture tools hosted at Geisinger Wyoming Valley Medical Center.^7^ The RA was available to answer questions and could input responses for the patient if requested. We enrolled a convenience sample of participants. Both the parent study and this supplement were reviewed and deemed exempt by the institutional review board of our healthcare system.

**Population Health Research Capsule**

What do we already know about this issue?

*Patient experience surveys are a feature of modern emergency care and used to determine reimbursement and sometimes physician bonuses and promotion*.

What was the research question?

*Were in-person surveys associated with a higher experience rating?*

What was the major finding of the study?

*All key questions favored the in-person vs mailed survey: “I felt informed” score 79.2 (262) vs 75.6 (265), P = .02; “I felt like my [doctor] took time to listen” 85.0 (261) vs 79.6 (266), P < .05*.

How does this improve population health?

*More accurate evaluation of ED patient experience can allow for meaningful improvement in ED care*.

### Measures

The parent survey asked, among other items, how people perceived their care with respect to time spent, feeling listened to, and overall satisfaction. Relevant survey questions are provided in the appendix. These differ slightly from the PG questions for reasons of copyright. Patients were asked to rank these values on a 1–5 Likert scale.

### Procedure

Using the data provided to our institution by the PG corporation, as well as local statistics kept for quality assurance, we determined the number of patients seen and discharged on the days for which we were enrolling, considering the relative exclusion criteria, and we used this number as the denominator for total people available. As part of the parent survey, we kept track of the response rate for those people who were specifically approached for enrollment. The PG data can be analyzed by the shift during which the patients were seen. Because we did not enroll patients overnight, we excluded data from those shifts.

Specific determination of our eligible patient denominator was as follows: Our department tracks numbers for total check-ins as well as admissions/observations and left before treatment complete (elopements, left without being seen, and against medical advice). We also track the ESI level 4 and 5 visits, which in our ED are seen almost exclusively APPS and, therefore, would have been excluded from the parent study. Although we didn’t enroll for the parent study overnight, we did not subtract the patients who checked in overnight as some of these may have waited and been seen by students in the morning.

### Analysis

We performed basic statistics using Microsoft Excel (Microsoft Corporation, Redmond, WA). Individual level data is not available from the PG surveys; however, they provide measures of central tendency including means and standard deviation for the quarter, which allowed us to perform statistical testing. Specifically, we used the mean and SD on from the PG surveys to perform Student *t*-statistic for the relevant questions. The PG survey converts the Likert scale into a 100-point score where 1 = 0, 2 = 25, 3 = 50, 4 = 75, and 5 = 100; therefore, we applied this conversion to our scale to give a final score for comparison.

## RESULTS

### Demographics

Demographic information is summarized in Table 1. The PG corporation does not provide demographic information for who completes their surveys but given that these are drawn from a similar population of patients, it is likely similar. Respondents were predominantly female (58%) and white (91%).

### Response Rate

During the study period from June–October 2022, a total of 625 patients were approached for enrollment in the parent study with 313 responses (rate 50.1%). Of the total 8,460 patients who were theoretically eligible on days we enrolled, this represents 3.70% of all possible patients. There was missing data on many of the surveys returned; only the ones with answers to the key questions were used for analysis. During the contemporaneous quarter, the hospital received a total of 266 responses to the PG survey during shifts 1 and 2 for an overall response rate of 3.14%.

### Patient Satisfaction

Results are summarized in Table 2. With respect to “I felt informed” the in-person score was 79.2 (of 262) vs 75.6 (265) for the mail in, *P* = .02. For “I felt like my [doctor] took time to listen” the in-person score was 85.0 (of 261) vs 79.6 (266) for the PG, *P* = .05. Finally, “satisfaction with care team” scored 83.0 in person of 263 vs 74.7 of 265 for the mail-in, *P* = 0.001.

## DISCUSSION

Patients rated their care more favorably when approached in person, and at a higher response rate. Although absolute numbers are important in terms of statistical and practical validity, the percentile rank is most important to hospital administration as this is the comparison they are held to for reimbursement.^1^,^2^ An absolute improvement of 5 points in score could lead to a 30-point increase in percentile rank, which is hugely significant for hospital administration, showing that these results have both statistical and real-world impact. However, given the current landscape of patient-experience survey collection, no hospital could unilaterally implement the in-person survey method to bolster their PG metrics. The value may lie in more robust data to inform incentives or more accurately provide feedback.

The in-person survey resulted in a greatly improved response rate in terms of people approached. Even in terms of overall numbers of responses, over the same amount of time there were a similar number of answers to the key questions returned for the in-person surveys despite the fact that the RAs typically only enrolled patients for 8–10 hours a day, weekdays only. Additionally, our overall response rate to the survey includes patients registered in the late evening and overnight who were not approached for enrollment; so, if someone were always available the expected response rate would be higher. Hospital Consumer Assessment of Healthcare Providers and Systems requires 300 surveys to be returned for the facility annually. The PG internal analysis states that 30–50 surveys are required to make comparisons between physicians, but for the ED as a whole this would provide a 50–55% confidence interval (CI). The number required to generate a 95% CI is ≈200 surveys.^2^

A positive patient experience is profitable. There is a strong correlation between negative patient experience and decreased revenue.^8^ Prior research efforts on the topic have shown that increasing the rating in the “Doctors” section evaluated here results in improvements in other domains as well.^9^ To this end, many organizations have attempted interventions such as scripting and customer service training, which require investment of physicians’ thinly stretched time, to improve their scores.^2^,^10^ Some studies of satisfaction used an in-person survey model and found increased response rates, consistent with our findings here.^10^

It is not possible to determine from this method whether the favorable response rate and ratings were the result of the human helping administer the survey vs the real-time delivery. Implementing an in-person system would be expensive, assuming dedicated personnel administered the survey. Our RAs were present in the department for an average of 8 hours/day, 5 days/week, over a 12-week period, representing 480 hours of work to enroll 313 people. Assuming a wage of $10/hour (minimum in our state is $7.25),^11^ this equates to $4,800 spent over the study period, or close to $20,000 annually. Put another way, each survey returned took 1.5 hours of labor. Many hospitals are investing in smart technology such as digital whiteboards; so, further research is needed to determine whether similar results would occur if these were used to deliver real-time surveys.

## LIMITATIONS

This study has several limitations. Importantly this was not a trial, and any conclusions are hypothesis-generating, at best. This also represents data from a single hospital and may not be generalizable. Because of the study design and the anonymous PG survey it is possible that different groups of people were answering each survey type. Different patients may have responded to different surveys, or possibly they decided not to return a mailed survey because they believed they had already completed one for the parent study. Both suffer from a low response rate. There is certainly a non-response bias in the in-person data, which favors more positive results for the in-person survey. However, completion of the mailed PG survey is subject to recall and multiple other types of bias^3^,^4^,^6^,^12^; however, the healthcare industry has accepted this as the way we evaluate our patients’ experience of their care. It is possible that the positive effect is not from eliminating recall bias but having a physical person administer the survey. Any future trial based on this study would do well to compare both. Regardless, as hospitals compete to “strive for 5” any potential advantage is worth using.

This study design did not have the ability to detect the effect of implicit or explicit bias on patients’ responses. Further investigation into this realm is needed. Our own internal quality control found evidence of non-response bias favoring more positive results. Multiple patients commented to the person enrolling that they didn’t want to answer the survey because they were dissatisfied with their care. As discussed above, this may mean that the positive results are from a theoretical representative of the hospital administering the survey rather than an effect of decreased recall bias. For individual hospitals seeking to gain an advantage, this is a desired effect. However, if applied more broadly, likely those systems with more resources will be able to devote the manpower to deliver an in-person survey to all discharged patients, favoring the already well-resourced hospitals.

## CONCLUSION

Patients administered an in-person survey answered the questions pertaining to the “Doctors” section on the Press Ganey survey rated their care more favorably than when they were mailed a survey to complete later, and with a higher response rate. Although this was not a trial and was subject to the limitations of survey studies and its design, the tool by which hospitals and ourselves as physicians are compared shares all these flaws with higher potential for non-response and recall bias. Using a real-time survey is another strategy that administrators may consider useful, as even small improvements in scores can lead to a dramatic change in percentile rank. A trial is needed to definitively answer this question. In this age of ED crowding and frustration interested leaders with the means to deploy such a tool should consider its use.

## Supplementary Information



## Figures and Tables

**Table 1:** Demographics of patients who completed the survey for the parent study.

Patient Age at Time of ED Visit	
Mean (SD)	49.5 (20.52)
Median (IQR)	47.0 (31.0, 66.0)
Range	18.0, 93.0
Missing	4
Patient Sex, n (%)	
Female	180 (58.6%)
Male	127 (41.4%)
Missing	4
Patient Racial Affiliation, n (%)	
Black	22 (7.2%)
White	280 (91.2%)
Other	3 (1.0%)
Unknown	2 (0.7%)
Missing	4
Patient Ethnicity, n (%)	
Non-Hispanic/Latino	285 (92.8%)
Hispanic/Latino	21 (6.8%)
Unknown	1 (0.3%)
Missing	4

*ED*, emergency department; *IQR*, interquartile range.

**Table 2:** Responses to survey questions delivered in-person versus by mail.

Question	In Person Survey Score (N)	Press Ganey Score (N)	*P-*value
I felt informed about my treatment and diagnosis after my visit.	79.2 (262)	75.57 (265)	0.02
I felt like my [doctor](s) took time to listen to me during my visit.	84.98 (261)	79.6 (266)	0.05
How would you rate your satisfaction with your care team on this visit?	83.03 (263)	74.67 (265)	0.001
