# Supplementary material for: Real-time Patient Experience Surveys Lead to Better Scores
Source: West J Emerg Med. 2025 Jun 25;26(4):810–4. doi: 10.5811/westjem.18713 (PMC12342542; doi:10.5811/westjem.18713)
Supplement: Supplementary file 1 [file wjem-26-810-s001.docx]

**Appendix 1. Survey Questions**

*In Person Survey*

Please select the answer that best reflects how you feel about the statement:

I felt informed about my treatment and diagnosis after my visit

1) Strongly disagree

2) Slightly disagree

3) Neutral

4) Slightly agree

5) Strongly agree

I felt like my provider(s) took time to listen to me during my visit

1) Strongly disagree

2) Slightly disagree

3) Neutral

4) Slightly agree

5) Strongly agree

How would you rate your satisfaction with your care team on this visit?

1) Highly dissatisfied

2) Somewhat dissatisfied

3) Neutral

4) Somewhat satisfied

5) Highly satisfied

*PressGaney®*

Your care was provided primarily by a doctor, physician assistant, nurse practitioner, or midwife. Please answer the following questions with that health care provider in mind

My doctors took time to listen

1) Very poor

2) Poor

3) Fair

4) Good

5) Very good

My doctor was informative regarding my treatment

1) Very poor

2) Poor

3) Fair

4) Good

5) Very good

Overall satisfaction with care team

1) Very poor

2) Poor

3) Fair

4) Good

5) Very good
